# Effects of blended microbial feed additives on performance, meat quality, gut microbiota and metabolism of broilers

**DOI:** 10.3389/fnut.2022.1026599

**Published:** 2022-12-06

**Authors:** Luli Zhou, Hui Li, Guanyu Hou, Chengjun Hu, Fengjie Ji, Weiqi Peng, Hanlin Zhou, Dingfa Wang

**Affiliations:** ^1^Tropical Crops Genetic Resources Institute, Chinese Academy of Tropical Agricultural Sciences, Haikou, China; ^2^College of Animal Science and Technology, Hainan University, Haikou, China; ^3^Zhanjiang Experimental Station, Chinese Academy of Tropical Agricultural Sciences, Zhanjiang, China

**Keywords:** blended microbial feed additives, broiler, meat quality, gut microflora, metabolism

## Abstract

The present study investigated the effects of blend microbial feed additive (BMFA) in diet on performance, meat quality, gut microbiota and metabolism of broilers. In this study 240 seventy-day-old female Wenchang broilers were randomly allocated into four groups with five replicates of 12 broilers each. Broilers in the control group was fed only basal diet (S0), and the other three groups were fed the same basal diet supplemented with 0.2% (S1), 0.4% (S2), or 0.6% (S3) of BMFA, respectively. The trial continued for 54 days. The results showed that broilers in S2 and S3 had lower average daily feed intake (ADFI) compared with S0 and S1 (*P* < 0.05). However, diet supplementation with BMFA had no significantly influence on the average daily gain (ADG) and the ratio of ADFI to ADG (F/G) (*P* > 0.05). The highest thigh muscle percentage was observed in S2 (*P* < 0.05) among all groups. Diet supplementation with BMFA reduced the shear force in both breast and thigh muscles (*P* < 0.05) of broilers. An increase (*P* < 0.05) in the total unsaturated fatty acid (USFA), monounsaturated fatty acids (MUFA), and ratio of unsaturated fatty acids to saturated fatty acid (USFA/SFA) in breast muscles was observed in S3 compared with S0. It was found that the S3 had a relatively higher abundance of *Lactobacillus* (*P* < 0.001), as well as a lower abundance of the *Bacteroides, Rikenellaceae RC9 gut group, Olsenella, Prevotellaceae UCG-001* and *Prevotella* (*P* < 0.05) than the S0. Correlation analysis indicated that a total of 17 differential metabolites between the S3 and S0 were significantly correlated with the 7 differential genera microflora. Overall, diet supplementation with 0.6% of BMFA can significantly improve the meat quality of broilers by decreasing the concentration of SFA and enhancing the levels of the total USFA, MUFA and USFA/SFA in breast muscles. Those findings were tightly bound to the higher proportion of *Lactobacillus* genus in the intestinal tract of broilers influenced by BMFA.

## Introduction

Broiler chickens are a great source of high-quality protein for human nutrition. Over the past several decades, antibiotics are widely used as an antimicrobial feed additive to enhance both animal health status and growth performance in conventional broiler production that has also driven the rapid development of broiler industry (1). Nevertheless, the application of antibiotics as feed additives in animal was forbidden gradually due to risk factors of cross-resistance and deposition in the tissues which affect the meat quality and human health in recent years (2). It is well-known that the intestine is not only a major organ responsible for the digestion and absorption of ingested dietary substances, it is also an effective physiological barrier against various pathogens infection (3). Thus, there is a demand urgently to find effective novel alternatives to antibiotics that could support gut health of broilers.

Probiotics are live microorganisms that plays potential beneficial roles in host according to the guidelines produced by FAO/WHO (4). Studies have shown that some probiotics, such as species of *Bifidobactrium, Enterococcus, Bacillus, Lactobacillus* and numerous yeast strains, have been applied to reconstitute the host intestinal microbiota, improve animal growth performance and strengthen immunity function (5–8). Nowadays, probiotics have developed into one of potential substitutes for antibiotics, and their application in livestock and poultry diets has become a research hotspot (9, 10).

The present study aimed to explore the effects of a blended microbial feed additive (BMFA) product (trade name: Mankouxiang) on the growth performance, carcass traits and meat quality of Wenchang broilers, and to investigate alterations of the intestinal microbiota (based on 16S rRNA sequencing) and cecal metabolites (based on chromatography-MS metabolomic techniques) as well as the correlation between intestinal flora and metabolism during BMFA treatment of broilers.

## Materials and methods

### Chemicals and reagents

Analytical-grade isopropanol and n-hexane were purchased from Sinopharm Chemical Reagent Co. (Shanghai, China). HPLC-grade methanol, acetonitrile, formic acid, ammonium acetate and ammonium hydroxide were supplied by Thermo Scientific (Waltham, MA, USA). HPLC-grade chloroform and pyridine were provided from Adamas-beta (Adamas, Shanghai, China). Trimethylsilyl diazomethane (with 2M in hexane, v/v) was obtained from Macklin (Macklin, Shanghai, China), fatty acid methyl esters (FAMEs) from Nu-Chek Prep, Inc. (Elysian, MN), methoxyamination hydrochloride from TCI Chemicals (Shanghai, China), adonitol from Sigma-Aldrich (St. Louis, MO, USA) and BSTFA (with 1% TMCS, v/v) from Regis Technologies (Morton Grove, IL, USA), respectively.

### Animals, experimental design and dietary formulation

All experimental procedures involving animal handling were reviewed and approved by the Institutional Animal Care and Use Committee of Chinese Academy of Tropical Agricultural Sciences (approval number CATAS-20201015-2).

A total of 240 seventy-day-old healthy female Wenchang broilers were randomly divided into four groups, each in 5 replicates (12 birds/replicate, 3 birds/cage). All broilers were housed in 3-tier wired cages for the 54-day feeding trial in the one same house (the size of each cage is 40 × 45 × 45 cm) and had free access to feed and water during the entire rearing period. The temperature of house was set at 25–28°C, and humidity percentage was maintained at 60–80%. The lighting program was set to produce 18 h of light and 6 h of darkness. The control group (S0) was fed the basal diet, while other three groups were fed the basal diet supplemented with blended microbial feed additives (BMFA) at 0.2% (S1), 0.4% (S2) or 0.6% (S3), respectively. The basal diet without antibiotic supplementation. The BMFA were acquired from Beijing Haoyouxuntian Biotechnology Limited Liability Company (Beijing, China). Each gram of the BMFA mainly contains 1.0 × 10^9^ CFU of *Enterococcus faecalis*, 1.0 × 10^9^ CFU of *Bacillus subtilis*, 1.0 × 10^9^ CFU of *Lactobacillus acidophilus*, and 1.0 × 10^9^ CFU of *Candida utilis* (according to its product manual). The dose of BMFA for this study were applied as recommended by manufacturer. The ingredients and calculated nutrient composition of basal diet are shown in Table 1.

**Table 1:** Ingredient and nutrient levels of basal diet (as-fed).

**Items**	**Contents**
**Ingredients (%)**
Corn	63.55
Fermented soybean meal, CP ≥46%	11.60
Middling, CP ≥15%	5.00
Corn gluten meal, CP ≥60%	5.00
Rapeseed meal, CP ≥38%	3.00
Rice bran meal, CP ≥15%	1.50
Soybean oil	7.00
Stone powder	0.50
Calcium hydrogen phosphate	0.40
Premix^a^	2.45
Total	100
**Nutrient levels**^**b**^ **(%)**	
Metabolic energy (MJ/kg)	13.96
Crude protein	15.32
Lysine	0.58
Methionine	0.27
Cysteine	0.27
Calcium	0.62
Available phosphorus	0.44

^a^Premix supplied the following per kilogram of diet: VA 3750 IU, VD_3_ 750 IU, VE 5 IU, VK_3_ 0.5 mg, Fe 30 mg, Cu 3 mg, Mn 24.5 mg, Zn 22 mg, I 0.5 mg, Se 0.3 mg. ^b^The nutrient levels are calculated values.

### Sample collection and measurements

Feed intake was recorded weekly, live weight and total feed consumption in each replicate were recorded at 1 and 54 d of the experimental period to determine average daily gain (ADG, g/d), average daily feed intake (ADFI, g/d) and feed-to-gain ratio (F/G). In addition, at the end of 54 day's experimental period, one bird per replicate was randomly selected and weighed after feed deprivation for 12 h. The selected birds were killed by exsanguination from the carotid artery. The yields of the slaughter, semi-eviscerated carcass, and eviscerated carcass were calculated as percentages of the live BW according to the regulations and requirements of Performance Ferms and Measurements for Poultry by the Ministry of Agriculture of the People's Republic of China (NY/T 823-2020), And then, breast muscle, thigh muscle, and abdominal fat were separated and weighed. The relative weights of breast muscle, thigh muscle, and abdominal fat were calculated as a percentage of the eviscerated carcass weight. Meanwhile, the cecum contents about 0.5 g were collected at the middle section, put into two sterile Eppendorf tubes, and stored at −80°C for subsequent 16S rRNA gene sequencing and metabolomic analysis. One side of the thigh and breast muscle (whole muscle tissue) were collected and stored at 4°C for meat quality trait analysis. About 2 g of tissue samples from another side of the thigh and breast muscle were collected and stored at −80°C for fatty acid composition analysis.

#### Meat quality

All measurements of meat quality traits were performed on thigh and breast muscle samples and the method used was similar to our previously study (11).

The pH value of breast and thigh muscle was measured at 45 min and 24 h postmortem using insertion of a pH meter (FG2, Shanghai, China) equipped with a penetrating electrode (Mettler Toledo, Changzhou, China). The pH meter was standardized with buffer solutions at pH 4.0 and 7.0 before assay.

Meat color was measured (average value of 3 measurements was taken from the middle and 2 corners of the muscle samples) using a hand-held Chromameter (CR 10, Konica Minolta INC., Osaka, Japan) and expressed as lightness (L^*^), redness (a^*^), and yellowness (b^*^) values.

The drip loss of each muscle sample (20 g) was analyzed at 24 h postmortem based on a bag method. The value of drip loss was calculated as a percentage of the weight loss over initial meat sample weight.

Instrumental tenderness was determined using the Warner-Bratzler test, assessing the resistance to shear force cut in cooked meat by a Texture Analyzer (Food Technology Corporation, Sterling, VA, USA).

#### Fatty acids composition

The fatty acid (FA) composition of meat was determined using a fatty acid methyl ester procedure. First, the muscle sample (50 mg) was weighed into an EP tube, and 1,000 μL of extract solution (isopropanol: n-hexane = 2:3, v/v) was added. After 30 s vortexing, each sample was ground for 4 min and homogenized by sonicating for 5 min in an ice-water bath. Then the homogenate was centrifuged at 12,000 rpm for 15 min at 4°C and the supernatant was collected. The above step was repeated twice, and the supernatants were combined. Supernatant (500 μL) was transferred into a new EP tube and vacuum dried. Subsequently, 1 mL of methanol and 1 mL of trimethylsilyl diazomethane were added, vortex and allowed to stand for 15 min at room temperature. Afterwards, the excess silylating reagent was evaporated by nitrogen blow down and the sample mixture dissolved in 2 mL of n-hexane. After centrifugation at 12,000 rpm for 10 min at 4°C, the supernatant was collected and transferred to the gas-phase vial. Finally, fatty acids were converted to the corresponding methyl ester and measured by gas chromatography-mass spectrometry (GC-MS) method.

The fatty acid methyl esters (FAMEs) were analyzed by Agilent 7890B GC with an Agilent 5977A mass spectrometer using an HP-5MS capillary column (30 m × 0.25 mm × 0.25 μm film thickness, Agilent, Palo Alto, USA) with the following temperature program: the initial oven temperature of 60°C was held for 2 min, increased to 150°C at 13°C/min, and then raised at 2°C/min to 230°C and held for 6 min. Besides, the inlet temperature was 230°C with constant flow of helium (He) gas at 0.8 mL/min in split less mode. MS detection mode was set as electron ionization (EI), scanning from 50 amu to 450 amu masses. Characteristic peaks were identified by comparing with the NIST 2014 Spectral Library and retention times of known analytical standards. Agilent Mass Hunter Software (QQQ) was used for quantitative analysis.

#### Microbiome 16S rRNA gene sequencing

Cecal content samples (100 mg) were extracted *via* methanol-mediated protein precipitation. Samples were then vortexed (5 min) followed by centrifugation (12,000 rpm for 10 min at 4°C). The procedure was repeated twice, and the supernatants were combined. Finally, 200 μL of supernatant was transferred to vial and used in the following analysis.

Total genome DNA from samples was extracted using CTAB/SDS method (12). DNA concentration and purity was monitored on 1% agarose gels. According to the concentration, DNA was diluted to 1 ng/μL using sterile water. The 16S rDNA gene of distinct regions (16S V3–V4) were amplified used specific primers 515F (5'-GTGCCAGCMGCCGCGGTAA-3') and 806R (5'-GGACTACHVGGGTWTCTAAT-3') with the barcode. Furthermore, the amplified PCR products were examined by gel electrophoresis (2% agarose), purified by using a Qiagen Gel Extraction Kit (Qiagen, Germany) to remove the unspecific DNA fragments and assessed by using the Qubit@ 2.0 Fluorometer (Thermo Scientific) and Agilent Bioanalyzer 2,100 system. At last, the products were pooled together with equal amount and sequenced on an Illumina NovaSeq platform and 250 bp paired-end reads were generated.

The raw reads obtained were filtered using the Trimmomatic program (https://www.usadellab.org/cms/?page=trimmomatic) and then merged using FLASH (version 1.2.7, http://ccb.jhu.edu/software/FLASH/). After that, quality control of the merged reads was performed using the QIIME (version 1.9.1, http://qiime.org/scripts/split_libraries_fastq.html) platform. The quality-filtered sequences were clustered into operational taxonomic units (OTUs) with a 97% similarity threshold using UPARSE (version 7.1, http://drive5.com/uparse/). Multiple sequence alignment was conducted using the MUSCLE software (Version 3.8.31, https://drive5.com/muscle). All the indices of alpha diversity, including Chao 1, Shannon, Simpson, Observed-species and Good-coverage, and the analysis of beta diversity were calculated with QIIME (version 1.9.1), and displayed with R software (version 2.15.3, http://www.r-project.org/).

#### Metabolomics analysis of cecal contents

Samples of cecal contents were analyzed using two analytical platforms, namely, gas chromatograph coupled with a time-of-flight mass spectrometer (GC-TOF-MS) and reverse-phase LC-MS/MS.

Samples were treated and analyzed following the previous published protocol with minor modification (13, 14). Briefly, 50 mg of sample of cecal content was put into a 2 ml EP tube, and two or three 5 mm steel ball was put in each sample tube. Add 1,000 μL extract (methanol: acetonitrile: water = 2:2:1, v/v) with internal standard ribonitol. Then the samples were homogenized at 35 Hz for 4 min and sonicated for 5 min in ice-water bath. Homogenization and sonication were repeated three times. Let the refrigerator stand at −40°C for 1 h. Centrifuge the sample at 4°C, 12,000 rpm for 15 min. Carefully transfer the 200 μL supernatant into a 1.5 mL EP tube. Take 50 μL of each sample and mix them into QC samples. Dry extract in vacuum concentrator. After evaporation in a vacuum concentrator, 60 μL of Methoxyamination hydrochloride (20 mg/mL in pyridine) was added and then incubated at 80°C for 30 min, then derivatized by 80 μL of BSTFA regent (1% TMCS, v/v) at 80°C for 1.5 h. Gradually cooling samples to room temperature, 5 μL of FAMEs (in chloroform) was added to QC sample. All samples were then analyzed by GC-MS.

GC-MS analysis was performed on a GCMS-QP2020 NX instrument, equipped with AOC-20 auto sampler and EI source (Shimazdu, Kyoto, Japan). The system utilized an Agilent DB-5MS capillary column (30 m × 250 μm I.D., 0.25 μm film thickness; J&W Scientific, Folsom, CA, USA). A 0.5 μL aliquot of sample was injected in split mode (5:1). Helium was used as the carrier gas, the front inlet purge flow was 3 mL/min, and the gas flow rate through the column was 1 mL/min. The initial temperature was kept at 50°C for 1 min, then raised to 310°C at a rate of 8°C/min, hold on 11.5 min. The injection, transfer line, and ion source temperatures were 280, 280, and 200°C, respectively. The energy was 70 eV in electron impact mode. The mass spectrometry data were acquired in full-scan mode with the m/z range of 50–500 after a solvent delay of 7.2 min.

Raw data analysis, including peak extraction, baseline adjustment, deconvolution, alignment and integration, was finished with Chroma TOF (version 4.3x, LECO) software and LECO-Fiehn Rtx5 database was used for metabolite identification by matching the mass spectrum and retention index (15). The cutoff for annotation was set at 0.3. Finally, a total of 312 peaks were detected and the peaks detected in less than half of QC samples or RSD >30% in QC samples was removed.

#### Metabolomics profiling by LC-MS/MS

Fifty mg of cecal content sample was weighted to an EP tube, and 1,000 μL extract solution (methanol: acetonitrile: water = 2: 2: 1, v/v/v, with isotopically-labeled internal standard mixture) was added. Then the samples were homogenized at 35 Hz for 4 min and sonicated for 5 min in ice-water bath. The homogenization and sonication cycle were repeated three times, followed by incubation at −40°C for 1 h and centrifugation for 15 min at 12,000 rpm and 4°C. The resulting supernatant was transferred to a fresh glass vial for analysis. The quality control (QC) sample was prepared by mixing an equal aliquot of the supernatants from all of the samples (16).

LC-MS/MS analysis was performed using a Vanquish UPLC system (Thermo Scientific, Waltham, MA, USA) coupled with an Orbitrap Q Exactive HFX mass spectrometer (Thermo Scientific, Waltham, MA, USA). An UPLC BEH Amide column (2.1 mm × 100 mm, 1.7 μm; Waters, Milford, MA, USA) was used for the UPLC-based separation of metabolites. The mobile phase consisted of solvent A (25 mM ammonium acetate and 25 mM ammonia hydroxide in water) and B (acetonitrile). The auto-sampler was conditioned at 4°C and the injection volume was 3 μL. In the information-dependent acquisition (IDA) mode, the acquisition software (Xcalibur, Thermo) continuously evaluated the full scan MS spectrum. The ESI source conditions were set as following: sheath gas flow rate as 30 arb, Aux gas flow rate as 25 arb, capillary temperature 350°C, full MS resolution as 60,000, MS/MS resolution as 7,500, collision energy as 10/30/60 in NCE mode, spray voltage as 3.6 kV (positive) or −3.2 kV (negative), respectively (16).

The raw data were converted to the mzXML format using ProteoWizard and processed with an in-house program, which was developed using R and based on XCMS, for peak detection, extraction, alignment, and integration. Then an in-house MS^2^ database (Biotree DB, Shanghai, China) was applied in metabolite annotation, and the cutoff for annotation was set at 0.3.

### Statistical analysis

Growth performance, slaughter and carcass traits and meat quality were analyzed by one-way analysis of variance (ANOVA) using SPSS 23.0 (for comparisons among multiple groups) (IBM-SPSS Inc., Chicago, IL, USA). The results in the tables were presented as mean and pooled SEM, and orthogonal polynomial contrasts (linear and quadratic) were used to evaluate the effect of dietary BMFA supplementation. Other figure results were shown with means ± SD. Significant differences were evaluated by Tukey multiple comparisons test at *P* < 0.05.

Principal component analysis (PCA) and supervised partial least-squares discriminant analysis (PLS-DA) were performed to visualize the metabolic differences between two groups. Moreover, *P*-values of metabolome analysis were calculated using the two-tailed Student's *t*-test and the value of fold change (FC) was calculated as the average normalized peak intensity ratio for comparisons between two groups. Metabolites with *P* < 0.05 and FC >1.10 or FC < 0.90 were considered statistically significant differential metabolites.

## Results

### Growth performance

Table 2 shows the effects of diet supplementation with BMFA on growth performance of the broilers. There were no differences in ADG, and F/G among groups (*P* > 0.05) during overall period. However, the ADFI of broilers in the S2 and S3 were lower compared with the S0 and S1 (*P* < 0.05), with a significant linearly decrease (*P* < 0.05).

**Table 2:** Effects of diets supplemented with BMFA on the growth performance of broilers.

**Items**	**Groups**				**SEM**	***P***	**Contrast** ^**a**^	
	**S0**	**S1**	**S2**	**S3**			***L***	***Q***
Initial weight, g	1107.65	1096.79	1102.36	1104.04	3.07	0.132	0.707	0.158
Final weight, g	2157.50	2179.38	2138.33	2171.33	16.67	0.355	0.995	0.743
Average daily gain (ADG), g	19.43	20.05	19.18	19.76	0.29	0.209	0.918	0.946
Average daily feed intake (ADFI), g	108.25^a^	108.46^a^	105.53^b^	106.46^b^	0.34	< 0.001	< 0.001	0.306
F/G (ADFI/ADG)	5.57	5.41	5.51	5.39	0.08	0.412	0.245	0.776

In the same row, values with no letter or the same letter superscripts mean no significant difference (*P* > 0.05), while with different letter superscripts mean significant difference (*P* < 0.05), *n* = 5. S0, blank control group, broilers were fed diet without BMFA supplementation; S1, S2, and S3, broilers were fed diet supplemented with 0.2, 0.4, and 0.6% of BMFA, respectively. ^a^*L*, linear; *Q*, quadratic.

### Slaughter performance

The slaughter performance of the broilers is presented in Table 3. No statistically significant differences were detected on semi-eviscerated carcass, eviscerated carcass, abdominal fat, or breast muscle percentage among the groups (*P* > 0.05). The dressing percentage in the S2 was lower compared to the S1 (*P* < 0.05), while there were no significant differences among the three groups (S0, S2, and S3) for dressing percentage (*P* > 0.05). Additionally, feeding broilers with diets containing BMFA resulted in an increase in both the breast and thigh muscle percentage compared to the S0. Especially, both the S1 and S2 had a significantly higher thigh muscle percentage than the S0 (*P* < 0.05), as well as significant linear (*P* < 0.05) and quadratic (*P* < 0.01) effects were observed for thigh muscle percentage.

**Table 3:** Effects of diets supplemented with BMFA on slaughter performance of broilers.

**Items**	**Groups**				**SEM**	***P***	**Contrast** ^**a**^	
	**S0**	**S1**	**S2**	**S3**			***L***	***Q***
Dressing percentage, %	92.97^ab^	93.42^a^	92.20^b^	93.21^ab^	0.27	0.027	0.686	0.304
Semi-eviscerated carcass percentage, %	83.23	85.33	83.98	86.15	1.05	0.238	0.135	0.975
Eviscerated carcass percentage, %	66.74	68.87	67.45	69.69	1.00	0.192	0.117	0.952
Abdominal fat percentage, %	10.07	9.41	9.01	8.68	0.59	0.402	0.101	0.780
Breast muscle percentage, %	13.40	15.31	15.07	15.27	0.27	0.164	0.085	0.211
Thigh muscle percentage, %	15.22^b^	17.70^a^	18.66^a^	16.94^ab^	0.58	0.005	0.030	0.002

In the same row, values with no letter or the same letter superscripts mean no significant difference (*P* > 0.05), while with different letter superscripts mean significant difference (*P* < 0.05), *n* = 5. S0, blank control group, broilers were fed diet without BMFA supplementation; S1, S2, and S3, broilers were fed diet supplemented with 0.2, 0.4, and 0.6% of BMFA, respectively. ^a^*L*, linear; *Q*, quadratic.

### Meat quality

As shown in Table 4, the dietary BMFA supplementation had no differences in the meat color, pH or drip loss of the thigh and breast muscle compared with the S0 (*P* > 0.05). Whereas, with BMFA supplementation increasing in the diet, the a^*^ (redness) value was linearly increased (*P* < 0.05), and the b^*^ (yellowness) value was linearly decreased (*P* < 0.05) in the thigh muscle. Our results showed that the shearing force values of thigh and breast muscles in the BMFA supplemented groups were lower than S0 (*P* < 0.05), and exhibited significant linear effects (*P* < 0.05).

**Table 4:** Effects of BMFA on meat quality of broilers.

**Items**	**Groups**				**SEM**	***P***	**Contrast** ^**a**^	
	**S0**	**S1**	**S2**	**S3**			***L***	***Q***
**Thigh muscle**
L*	37.92	40.87	38.68	38.78	0.79	0.274	0.938	0.198
a*	14.22	16.20	17.65	17.56	0.46	0.152	0.037	0.372
b*	13.28	12.94	10.72	10.22	0.41	0.094	0.018	0.934
pH_45min_	6.48	6.45	6.39	6.42	0.06	0.942	0.630	0.771
pH_24h_	5.80	5.95	5.99	6.00	0.01	0.178	0.051	0.336
Drip loss (%)	7.23	6.37	7.11	6.84	0.27	0.212	0.354	0.204
Shear force (N)	32.25^a^	18.08^c^	18.00^c^	23.67^b^	0.59	0.000	0.000	0.000
**Breast muscle**
L*	49.10	50.24	49.62	51.84	0.48	0.271	0.107	0.595
a*	4.20	4.34	4.36	4.19	0.09	0.853	0.992	0.391
b*	18.48	14.78	15.38	15.25	0.23	0.198	0.053	0.894
pH_45min_	6.32	6.09	6.12	6.07	0.10	0.616	0.287	0.550
pH_24h_	5.55	5.52	5.51	5.50	0.01	0.738	0.291	0.795
Drip loss (%)	4.92	3.96	4.94	4.64	0.35	0.248	0.612	0.196
Shear force (N)	13.75^a^	8.63^b^	6.63^b^	6.25^b^	0.12	0.000	0.000	0.004

In the same row, values with no letter or the same letter superscripts mean no significant difference (*P* > 0.05), while with different letter superscripts mean significant difference (*P* < 0.05), n = 5. S0, blank control group, broilers were fed diet without BMFA supplementation; S1, S2, and S3, broilers were fed diet supplemented with 0.2, 0.4, and 0.6% of BMFA, respectively. ^a^*L*, linear; *Q*, quadratic. L*, a*, b* represent lightness, redness, and yellowness, respectively.

### Fatty acids composition

As displayed in Table 5, fatty acid composition of the thigh muscle did not differ among all groups (*P* > 0.05). Furthermore, it was found that level of C16:1n7 increased linearly (*P* < 0.05), whereas level of C22:6n3 decreased linearly (*P* < 0.05) in the thigh muscle with increasing BMFA concentration in the diet.

**Table 5:** Fatty acids profile in the thigh muscle of broilers (%).

**Items**	**Group**				**SEM**	***P***	**Contrast** ^**a**^	
	**S0**	**S1**	**S2**	**S3**			***L***	***Q***
C6:0	3.63	2.96	2.38	2.57	0.56	0.424	0.124	0.943
C8:0	2.15	1.75	1.40	1.53	0.33	0.424	0.125	0.918
C10:0	2.54	3.54	2.99	2.60	0.85	0.827	0.807	0.386
C11:0	3.90	3.18	2.54	2.78	0.62	0.446	0.136	0.928
C14:0	13.04	18.05	11.58	14.12	4.99	0.818	0.936	0.612
C16:0	0.65	0.65	0.51	0.55	0.08	0.569	0.318	0.713
C17:0	3.72	3.93	3.49	3.53	0.75	0.973	0.844	0.766
C18:0	4.83	4.88	4.32	3.75	0.86	0.773	0.450	0.483
C20:0	0.15	0.11	0.10	0.11	0.02	0.574	0.182	0.816
C21:0	0.14	0.12	0.12	0.13	0.02	0.890	0.576	0.720
C22:0	0.89	0.72	0.58	0.63	0.14	0.430	0.128	0.906
C14:1n5	0.96	0.88	0.78	0.80	0.09	0.480	0.154	0.901
C15:1n5	0.22	0.19	0.16	0.17	0.03	0.520	0.169	0.936
C16:1n7	9.06	11.25	11.95	12.17	1.15	0.248	0.049	0.907
C17:1n7	0.16	0.15	0.14	0.14	0.01	0.632	0.240	0.903
C18:1n9	6.65	6.51	8.78	8.23	2.22	0.851	0.549	0.759
C18:1n9c	10.25	9.41	10.56	10.41	2.13	0.981	0.957	0.769
C20:1n9	0.10	0.12	0.11	0.12	0.01	0.451	0.234	0.789
C22:1n9	0.28	0.24	0.19	0.20	0.04	0.435	0.131	0.956
C18:2n6t	9.34	10.06	14.78	14.30	3.34	0.561	0.251	0.644
C18:2n6c	12.49	8.15	10.60	10.15	2.98	0.785	0.531	0.523
C18:3n6	5.83	6.57	5.60	5.19	1.40	0.914	0.814	0.537
C20:2	0.84	0.69	0.55	0.60	0.12	0.411	0.123	0.942
C20:3n6	0.98	0.81	0.65	0.71	0.15	0.455	0.138	0.928
C20:4n6	3.76	2.98	2.44	2.67	0.50	0.297	0.077	0.824
C20:5n3	1.87	1.01	1.84	0.96	0.40	0.226	0.220	0.907
C22:2n6	0.69	0.53	0.43	0.47	0.10	0.309	0.079	0.775
C22:6n3	0.88	0.55	0.44	0.41	0.14	0.104	0.016	0.852
SFA	35.64	39.91	30.00	32.28	5.89	0.665	0.621	0.501
USFA	64.36	60.09	70.00	67.72	5.89	0.665	0.621	0.501
MUFA	27.69	28.75	32.67	32.25	3.59	0.699	0.321	0.748
PUFA	36.67	31.35	37.33	35.47	3.62	0.655	0.848	0.438
USFA/SFA	2.24	1.79	2.74	2.36	0.55	0.674	0.755	0.586

In the same row, values with no letter or the same letter superscripts mean no significant difference (*P* > 0.05), while with different letter superscripts mean significant difference (*P* < 0.05), *n* = 5. S0, blank control group, broilers were fed diet without BMFA supplementation; S1, S2, and S3, broilers were fed diet supplemented with 0.2, 0.4, and 0.6% of BMFA, respectively. ^a^*L*, linear; *Q*, quadratic.

Noteworthy, the composition of fatty acid in breast muscle tissue was greatly affected by dietary BMFA supplementation (Table 6). Broilers in the group fed with 0.6% BMFA (S3), overall level of SFA (mainly include C6:0, C8:0, C11:0, C12:0, C14:0, C20:0, and C22:0) (*P* < 0.01) of breast muscle decreased significantly, whereas the levels of the total USFA (largely contain C16:1n7, C18:1n9, C18:2n6c, and C18:3n6) (*P* < 0.01), MUFA (predominantly involve C16:1n7 and C18:1n9) (*P* < 0.001) and USFA/SFA (*P* < 0.01) of breast muscle showed a significant increase compared to the S0. Meanwhile, with an increasing amount of added BMFA resulting in a significant linear effect of the total SFA (*P* < 0.01), USFA (*P* < 0.01), MUFA (*P* < 0.001) and USFA/SFA (*P* < 0.01).

**Table 6:** Fatty acids profile in the breast muscle of broilers (%).

**Items**	**Group**				**SEM**	***P***	**Contrast** ^**a**^	
	**S0**	**S1**	**S2**	**S3**			***L***	***Q***
C6:0	4.68^a^	4.78^a^	2.83^b^	2.77^b^	0.37	0.001	0.001	0.025
C8:0	2.73^a^	2.96^a^	1.78^b^	1.64^b^	0.24	0.002	0.005	0.015
C10:0	6.23	5.51	9.94	11.64	3.03	0.443	0.245	0.306
C11:0	4.90^a^	5.13^a^	3.04^b^	2.73^b^	0.43	0.002	0.002	0.015
C12:0	2.31^a^	2.44^a^	1.44^b^	1.29^b^	0.21	0.002	0.003	0.016
C14:0	25.68^a^	26.31^a^	19.72^a^	6.06^b^	3.50	0.003	0.005	0.004
C16:0	0.91^a^	0.81^a^	0.52^b^	0.72^ab^	0.07	0.005	0.007	0.704
C17:0	2.17^b^	2.59^ab^	2.31^b^	3.50^a^	0.29	0.022	0.025	0.097
C18:0	3.36	3.83	3.58	4.76	0.46	0.195	0.113	0.294
C20:0	0.16^ab^	0.19^a^	0.12^bc^	0.09^c^	0.01	0.001	0.011	0.000
C21:0	0.18^ab^	0.20^a^	0.13^b^	0.13^b^	0.02	0.008	0.034	0.017
C22:0	1.12^a^	1.18^a^	0.69^b^	0.62^b^	0.10	0.002	0.003	0.016
C14:1n5	1.17^ab^	1.31^a^	0.97^ab^	0.89^b^	0.09	0.018	0.058	0.018
C15:1n5	0.27^ab^	0.35^a^	0.25^b^	0.21^b^	0.03	0.013	0.213	0.005
C16:1n7	8.96^b^	10.77^a^	14.37^a^	15.23^a^	0.78	0.000	0.000	0.051
C17:1n7	0.20^ab^	0.22^a^	0.18^ab^	0.16^b^	0.01	0.041	0.107	0.019
C18:1n9	4.45	3.92	4.60	6.53	0.73	0.116	0.170	0.058
C18:1n9c	4.96^c^	7.18^b^	10.75^ab^	12.03^a^	0.75	0.000	0.000	0.075
C20:1n9	0.13	0.15	0.12	0.13	0.01	0.102	0.403	0.082
C22:1n9	0.36^a^	0.38^a^	0.23^b^	0.21^b^	0.03	0.002	0.002	0.017
C18:2n6t	9.15	4.12	7.40	9.06	2.35	0.418	0.738	0.123
C18:2n6c	3.46^b^	1.82^b^	4.90^ab^	7.68^a^	1.04	0.007	0.035	0.004
C18:3n6	2.22^b^	2.85^b^	3.33^b^	5.70^a^	0.58	0.003	0.003	0.017
C20:2	1.04^a^	1.11^a^	0.69^b^	0.61^b^	0.09	0.003	0.005	0.015
C20:3n6	1.22^a^	1.29^a^	0.86^ab^	0.73^b^	0.12	0.008	0.010	0.025
C20:4n6	4.67^a^	4.74^a^	2.86^b^	2.55^b^	0.40	0.001	0.001	0.020
C20:5n3	1.75^a^	1.78^a^	1.11^b^	1.09^b^	0.15	0.004	0.004	0.050
C22:2n6	0.81^ab^	0.96^a^	0.58^b^	0.56^b^	0.07	0.004	0.027	0.010
C22:6n3	0.75	1.11	0.68	0.69	0.15	0.202	0.838	0.091
SFA	54.42^a^	55.94^a^	46.12^ab^	35.94^b^	2.93	0.001	0.001	0.002
USFA	45.58^b^	44.06^b^	53.88^ab^	64.06^a^	2.85	0.001	0.001	0.002
MUFA	20.50^b^	24.92^b^	31.47^a^	35.39^a^	1.57	0.000	0.000	0.017
PUFA	25.08	19.14	22.41	28.67	2.52	0.119	0.768	0.020
USFA/SFA	0.86^b^	0.80^b^	1.20^b^	1.90^a^	0.17	0.001	0.003	0.002

In the same row, values with no letter or the same letter superscripts mean no significant difference (*P* > 0.05), while with different letter superscripts mean significant difference (*P* < 0.05), *n* = 5. S0, blank control group, broilers were fed diet without BMFA supplementation; S1, S2, and S3, broilers were fed diet supplemented with 0.2, 0.4, and 0.6% of BMFA, respectively. ^a^*L*, linear; *Q*, quadratic.

### Microbiome 16S rRNA sequencing

Alpha-diversity measures revealed cecal bacterial diversity (Shannon and Simpson indexes) and richness (ACE and Chao1 indexes) did not differ between the S0 and S3 (*P* > 0.05) (Supplementary Table 1). In addition, the beta diversity analysis was performed using principal coordinates analysis (PCoA), and it exhibited that the variations of the microbial community composition were explained by PC1 (61.26%) and PC2 (12.15%), accounting for a total of 73.41% of the overall diversity between the S0 and S3 (Supplementary Figure 1).

**Figure 1:**
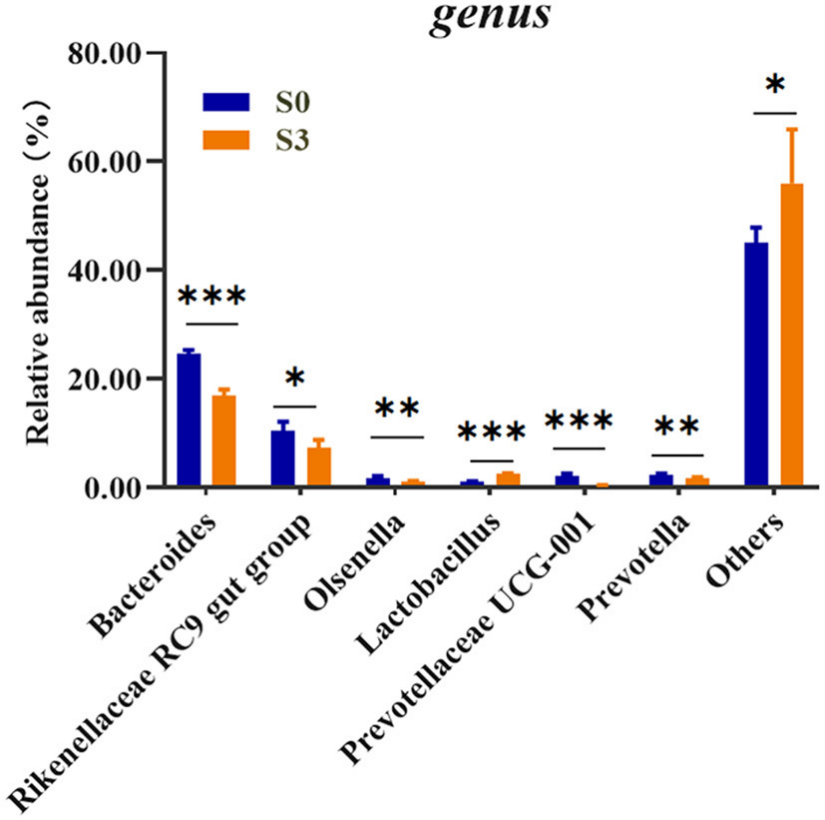
Comparison of relative abundances of significantly different microbial taxa at the genus levels between S0 and S3. S0, blank control group, broilers were fed diet without BMFA supplementation; S3, broilers were fed diet supplemented with 0.6% of BMFA. The asterisk (*) indicates *P* < 0.05, (**) indicates *P* < 0.01, and (***) indicates *P* < 0.001, *n* = 5.

To assess the microbial community alterations, the relative abundance at different ranking levels from phylum to genus were compared and profiled between the S0 and S3 (Supplementary Table 2). The result showed a dramatic difference in microbiota composition between the two groups at the genus level (Figure 1). It was found that the S3 had a relatively higher abundance of *Lactobacillus* (*P* < 0.001) and Others (*P* < 0.05), as well as a lower abundance of the *Bacteroides* (*P* < 0.001), *Rikenellaceae RC9 gut group* (*P* < 0.05), *Olsenella* (*P* < 0.01), *Prevotellaceae UCG-001* (*P* < 0.001) and *Prevotella* (*P* < 0.01) than in the S0.

### Metabolomics analysis of cecal content

To further investigate the changes of the global metabolite profiles in the cecal of broilers given BMFA addition, the samples between the S0 and S3 were combined for PCA and PLS-DA. The unsupervised PCA model for the metabolite dataset between the S0 and S3 was built based on two principal components- PC1 and PC2, contributing to 27.4 and 17.4% percentages of the variation, respectively (Supplementary Figure 2A). Moreover, the supervised PLS-DA model of metabolite profiles showed a segregation between the S0 and S3 (Supplementary Figure 2B).

There were seventeen significantly different metabolites detected in the S3 vs. the S0, with four metabolites upregulated and fourteen metabolites downregulated (Supplementary Table 3). Then all these differential metabolites were further used for pathway enrichment analysis by MetaboAnalyst 5.0 (https://www.metaboanalyst.ca/). Finally, the results demonstrated that a total of six metabolic pathways were enriched, including biotin metabolism, tryptophan metabolism, carnitine synthesis, lysine degradation, steroidogenesis and tyrosine metabolism (Supplementary Figure 3). Notably, five differential metabolites were found to be involved in these above potential metabolic pathways (Table 7).

**Table 7:** Metabolic pathways for differential metabolites in the S3 compared with the S0.

**Num**.	**Differential metabolites**	***P*-value**	**FC**	**Metabolic pathways**
1	L-lysine	0.007	0.52	Biotin metabolism; Carnitine synthesis; Lysine degradation
2	Indoleacetic acid	0.032	0.46	Tryptophan metabolism
3	Kynurenic acid	0.036	0.52	Tryptophan metabolism
4	Tetrahydrocorticosterone	0.037	0.48	Steroidogenesis
5	Pyrocatechol	0.040	0.29	Tyrosine metabolism

### Correlation analysis

**Figure 2:**
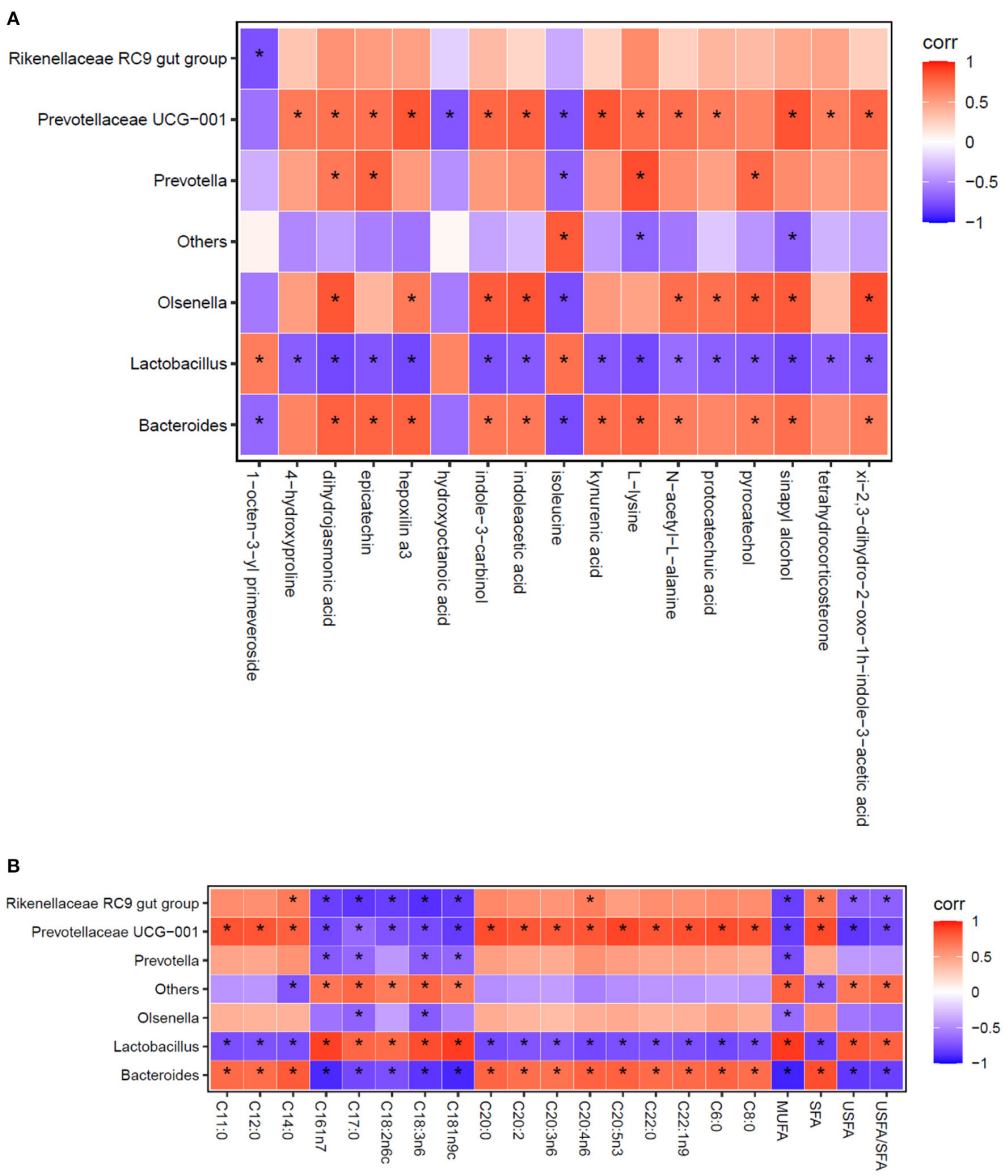
Spearman correlation analysis between the relative abundance of the most abundant cecal microbes at the genus level and cecal metabolites **(A)**, as well as the composition of fatty acid in breast muscles **(B)**. Each column represents a differential metabolite **(A)** or fatty acid **(B)**, each row represents a genus, and each square represents a correlation coefficient between the corresponding microbiota component and metabolite or fatty acid. The cross-correlation coefficients were normalized in the range of −1 to 1. Red (1) represent highest values whereas blue (−1) represent the lowest values. The asterisk (*) indicates *P* < 0.05.

Using the Spearman statistical analysis method, the S3 vs. S0 group had the clear distinction between differential flora at the genus level and differential metabolites in cecal digesta of broilers (Figure 2A), and between differential flora at the genus level and fatty acid composition in breast muscle of broilers (Figure 2B).

As shown in Figure 2A, the analysis revealed that a total of 17 differential metabolites between the S3 and S0 were significantly correlation with the 7 differential genera. When combined with data presented in Table 7 and Figure 2A reveals several important observations. It was found that two differential metabolites, indoleacetic acid and kynurenic acid in the tryptophan metabolism pathway were significantly positive correlated with *Bacteroides* and *Prevotellaceae UCG-001* (*P* < 0.05) and negative correlated with *Lactobacillus* (*P* < 0.05). Additionally, L-lysine was not only involved in metabolic pathways of biotin metabolism, carnitine synthesis and lysine degradation but also significantly positive correlated with *Bacteroides, Prevotellaceae UCG-001*and *Prevotella* (*P* < 0.05), and was negative correlated with *Lactobacillus* (*P* < 0.05). Furthermore, pyrocatechol in the pathway of tyrosine metabolism showed a positive correlation with *Bacteroides, Olsenella* and *Prevotella* (*P* < 0.05), but a negative correlation with *Lactobacillus* (*P* < 0.05). Moreover, tetrahydrocorticosterone that is directly associated with steroidogenesis metabolic pathway is positively related to the abundance of *Prevotellaceae UCG-001* (*P* < 0.05) and negatively related to the abundance of *Lactobacillus* (*P* < 0.05).

As shown in Figure 2B, the results exhibited that *Lactobacillus, Bacteroides* and *Prevotellaceae UCG-001* were the most important representative genera that can influence the compositional changes of fatty acid in breast muscle of broilers.

## Discussion

BMFA is a mixture of multi-microbe probiotics which mainly consist of *Enterococcus faecalis, Bacillus subtilis, Lactobacillus acidophilus* and *Candida utilis*. It is generally known that the beneficial influences of probiotics are mainly achieved by changing the intestinal microbiota structure, increasing the population of beneficial microorganisms and inhibiting the proliferation of bacterial pathogens (17).

The results of our present study indicated that compared with the control, dietary BMFA supplementation with 0.4% or 0.6% significant decreased ADFI while did not appear to effect on ADG, which leading to a trend toward reduction in F/G. In addition, dietary BMFA addition with 0.2–0.6% also effectively increase the percentages of breast and thigh muscles but reduced abdominal fat rate. All these findings revealed that BMFA positively affect growth and slaughter performance of broilers.

The present study indicated that diet addition with 0.6% of BMFA (S3) can better improve the meat quality and flavor of Wenchang chicken. Previous studies showed that the gut microbiota has a large influence on host metabolism, and the cecum is colonized with a sufficient number of readily harvestable microbiota (18, 19). Thus, the cecal content was selected for sampling, and an integrated gut microbiome and metabolomic analysis was carried out between the S0 and S3 to further investigate the effects of BMFA on broiler intestinal metabolism. It was found that BMFA-supplemented diets affected the cecum microbiota structure considerably at different levels including phylum, class, order, family and genus. Specially, at the genus level, BMFA-supplemented diets significantly increased the abundance of beneficial microorganisms like *Lactobacillus*, and reduced the abundances of harmful microorganisms, such as the pro-inflammatory bacteria *Prevotella*. Meanwhile, the abundances of *Lactobacillus* and *Prevotella* significant negatively or positively correlation with downregulation of the levels of L-lysine and pyrocatechol, respectively, and in turn predominantly impacted metabolic pathways of lysine degradation, biotin metabolism, carnitine synthesis and tyrosine metabolism.

Remarkably, downregulation of the pyrocatechol was accompanied by an uptrend of the norepinephrine (*P* = 0.445, FC = 1.25) in the S3 compared to the S0. So, it can be speculated that norepinephrine may not be converted to pyrocatechol due to the inhibition of the catechol *O*-methyltransferase activity by the presence of dietary with 0.6% BMFA, and this process might trigger suppression of appetite in broilers, ultimately resulting in the markedly decrease in ADFI of broilers (20, 21). Additionally, the results showed that the level of *L*-lysine was significantly downregulated in the S3 compared to the S0. This might because *L*-lysine, as a precursor for carnitine synthesis, is participated in the synthesis of carnitine, and already synthesized carnitine is further involved in the process of protein synthesis and transports fatty acids into mitochondria to supply energy that could lead to improvement in growth and slaughter performance of broilers (22, 23).

As is known that tenderness, assessed by shear force, is one of the most important attributes of meat quality (24). In this study, the breast and thigh muscles of broilers fed the diet supplemented with 0.2–0.6% of BMFA had a significantly lower shear force than that of broilers in the S0, which improved the muscle tenderness. It was reported that the low values for shear force might be related to the high content of proteins in the muscle (25). Also, isoleucine is a branched-chain amino acid which act as an important substrate in protein synthesis (26). Notably, we observed that the level of isoleucine in the S3 was significantly higher than in the S0, and implied that the higher level of isoleucine stimulated muscle protein synthesis of broilers, resulting in the improvement of meat tenderness of broilers.

Furthermore, although BMFA-supplemented diets have no effect on fatty acid composition of the leg muscles of broilers, it appears that the fatty acid composition of the breast muscles was significant influenced by BMFA. It means that BMFA could contribute to better the flavor of breast meat by significantly both increasing USFA relative content and decreasing that of SFA. Meanwhile, further microbiota analysis between the S3 and S0 groups showed that BMFA led to a dramatic decrease of some genera including *Bacteroides, Rikenellaceae RC9 gut group, Olsenella, Prevotellaceae UCG-001* and *Prevotella*, and strongly increased in the abundance of *Lactobacillus*. Presumably, lactic acid, produced by *Lactobacillus*, can reduce the intestinal pH, and has a strong inhibiting effect on other genera in an acidic environment (27), that is, the higher proportion of *Lactobacillus* genus enable its members to occupy a wide range of ecological niches in the intestinal tract, and finally play a critical role as a good antioxidant in protecting the USFA from oxidation (28). On the other hand, higher abundance of the genus *Lactobacillus* in the S3 could produce more ligands of aryl hydrocarbon receptor (AhR) than the S0 (29). However, the results presented that several tryptophan metabolites as ligands for AhR (30, 31), such as kynurenic acid, indoleacetic acid, xi-2,3-dihydro-2-oxo-1h-indole-3-acetic acid and indole-3-carbinol, were significantly downregulated in the S3 compared to the S0. We reason that this seemingly conflictive results might be due to the binding of these tryptophan metabolites and AhR. Then, the activated AhR probably not only inhibited the activity of fatty acid synthase, resulting in a decrease in SFA (32), but also exerted an important role in regulation of intestinal immunity, inflammation as well as maintenance of gut homeostasis (33).

In addition, the level of tetrahydrocorticosterone in the metabolic pathway of steroidogenesis was significantly downregulated in the S3 compared with the S0, and suggested BMFA could effectively reduce production of the reactive oxygen species (ROS) and then relieve the oxidative stress in the gut (34, 35).

## Conclusions

Overall, dietary supplementation with 0.2–0.6% of BMFA could improve some slaughter parameters and fatty acids profile of broiler meat without affecting growth performance, which closely associated with alteration in gut microbiota and its metabolites influenced by BMFA.

## Data availability statement

The raw sequence datasets in this study can be found in online repositories. The names of the repository/repositories and accession number(s) can be found below: https://www.ncbi.nlm.nih.gov/, PRJNA876778. The other datasets during and/or analyzed during the current study are available from the corresponding author on reasonable request.

## Ethics statement

All procedures involving animal care and management were approved by the Institutional Animal Care and Use Committee of Chinese Academy of Tropical Agricultural Sciences (approval number CATAS-20201015-2).

## Author contributions

DW and HZ designed the experiments. LZ, HL, CH, FJ, WP, and GH conducted the experiments. LZ and HL analyzed the data. LZ wrote the main manuscript. DW reviewed and edited the manuscript. All authors read and approved the final manuscript.

## Funding

This study was financially supported by the Key Research and Development Project of Hainan Province, China (No. ZDYF2021SHFZ261) and Wenchang Chicken Superiority Characteristic Industrial Cluster Project of Hainan Province, China (No. WCSCICP2022-06).

## Conflict of interest

The authors declare that the research was conducted in the absence of any commercial or financial relationships that could be construed as a potential conflict of interest.

## Publisher's note

All claims expressed in this article are solely those of the authors and do not necessarily represent those of their affiliated organizations, or those of the publisher, the editors and the reviewers. Any product that may be evaluated in this article, or claim that may be made by its manufacturer, is not guaranteed or endorsed by the publisher.
