# Supplementary material for: Effects of blended microbial feed additives on performance, meat quality, gut microbiota and metabolism of broilers
Source: Front Nutr. 2022 Dec 6;9:1026599. doi: 10.3389/fnut.2022.1026599 (PMC9764441; doi:10.3389/fnut.2022.1026599)
Supplement: Supplementary file 1 [file Data_Sheet_1.docx]

**Supplementary Tables and Figures**

**Table S1** Alpha diversity indexes inter-group difference table

| Items | Groups | | SEM | *P* |
| --- | --- | --- | --- | --- |
|  | S0 | S3 |  |  |
| observed_species | 716.00 | 707.20 | 36.79 | 0.884 |
| shannon | 6.95 | 7.16 | 0.16 | 0.426 |
| simpson | 0.98 | 0.99 | 0.01 | 0.273 |
| chao1 | 821.38 | 811.65 | 45.59 | 0.896 |
| ACE | 830.98 | 814.51 | 42.06 | 0.811 |
| goods_coverage | 99.52 | 99.56 | 0.04 | 0.545 |

S0, the broilers were fed a basal diet; S3, the broilers were fed a basal diet supplementation with 0.6 % of BMFA. n=5.

**Table S2** Relative abundance of the cecal microbial community (within the top 10 and others) from the S0 and S3 at different levels

| Name | Groups | | SEM | *P* |
| --- | --- | --- | --- | --- |
|  | S0 | S3 |  |  |
| At the plylum level | | | | |
| *Bacteroidota* | 55.77 | 49.92 | 1.96 | 0.142 |
| *Firmicutes* | 29.54 | 33.55 | 1.54 | 0.212 |
| *Actinobacteriota* | 4.41 | 2.25 | 0.75 | 0.158 |
| *Proteobacteria* | 2.04 | 2.03 | 0.50 | 0.993 |
| *Euryarchaeota* | 1.47 | 1.59 | 0.54 | 0.915 |
| *Verrucomicrobiota* | 0.56 | 2.43 | 0.47 | 0.039 |
| *Desulfobacterota* | 1.59 | 1.32 | 0.13 | 0.326 |
| *Cyanobacteria* | 0.21 | 0.23 | 0.11 | 0.942 |
| *Campilobacterota* | 0.10 | 0.41 | 0.07 | 0.023 |
| *unidentified_Bacteria* | 2.68 | 4.66 | 0.87 | 0.277 |
| Others | 1.63 | 1.62 | 0.09 | 0.959 |
| At the class level | | | | |
| *Bacteroidia* | 55.77 | 49.92 | 1.96 | 0.142 |
| *Clostridia* | 23.45 | 24.13 | 0.62 | 0.617 |
| *Bacilli* | 3.43 | 5.74 | 0.70 | 0.100 |
| *Coriobacteriia* | 2.41 | 1.76 | 0.19 | 0.076 |
| *Gammaproteobacteria* | 1.61 | 1.34 | 0.11 | 0.213 |
| *Methanobacteria* | 0.94 | 0.92 | 0.06 | 0.897 |
| *Desulfovibrionia* | 0.92 | 2.59 | 0.32 | 0.001 |
| *Verrucomicrobiae* | 0.68 | 3.00 | 0.44 | 0.001 |
| *Negativicutes* | 2.94 | 2.01 | 0.22 | 0.026 |
| *unidentified_Bacteria* | 1.31 | 1.18 | 0.41 | 0.883 |
| Others | 5.35 | 5.36 | 0.46 | 0.991 |
| At the order level | | | | |
| *Bacteroidales* | 55.28 | 49.02 | 2.06 | 0.134 |
| *Lachnospirales* | 10.53 | 9.56 | 0.63 | 0.479 |
| *Oscillospirales* | 8.95 | 10.80 | 0.71 | 0.209 |
| *Coriobacteriales* | 3.48 | 1.51 | 0.78 | 0.224 |
| *Lactobacillales* | 1.97 | 4.14 | 0.70 | 0.127 |
| *Erysipelotrichales* | 0.81 | 3.03 | 0.57 | 0.044 |
| *Methanobacteriales* | 1.47 | 1.59 | 0.54 | 0.915 |
| *Desulfovibrionales* | 0.71 | 3.17 | 0.49 | 0.003 |
| *Clostridiales* | 1.00 | 0.79 | 0.48 | 0.840 |
| *Verrucomicrobiales* | 0.67 | 3.00 | 0.44 | 0.001 |
| Others | 15.60 | 14.61 | 1.10 | 0.677 |
| At the family level | | | | |
| *Bacteroidaceae* | 27.49 | 19.06 | 1.98 | 0.021 |
| *Rikenellaceae* | 12.06 | 10.05 | 0.77 | 0.212 |
| *Lachnospiraceae* | 10.52 | 9.56 | 0.63 | 0.479 |
| *Prevotellaceae* | 5.44 | 5.94 | 0.92 | 0.803 |
| *Muribaculaceae* | 4.02 | 8.08 | 0.74 | 0.000 |
| *Ruminococcaceae* | 6.38 | 7.43 | 0.58 | 0.393 |
| *Atopobiaceae* | 3.01 | 0.83 | 0.77 | 0.170 |
| *Lactobacillaceae* | 1.53 | 3.87 | 0.70 | 0.094 |
| *Erysipelotrichaceae* | 0.50 | 2.70 | 0.57 | 0.046 |
| *Methanobacteriaceae* | 1.47 | 1.59 | 0.54 | 0.915 |
| Others | 28.24 | 31.46 | 1.75 | 0.388 |
| At the genus level | | | | |
| *Bacteroides* | 24.63 | 16.92 | 1.32 | 0.000 |
| *Rikenellaceae RC9 gut group* | 10.42 | 7.33 | 0.71 | 0.017 |
| *Olsenella* | 1.83 | 1.09 | 0.15 | 0.004 |
| *Lactobacillus* | 1.20 | 2.61 | 0.24 | 0.000 |
| *Faecalibacterium* | 3.85 | 4.13 | 0.21 | 0.553 |
| *Methanobrevibacter* | 2.09 | 1.93 | 0.47 | 0.880 |
| *Prevotellaceae* *UCG-001* | 2.20 | 0.40 | 0.31 | 0.000 |
| *Alistipes* | 1.33 | 1.91 | 0.17 | 0.082 |
| *CHKCI001* | 2.47 | 1.94 | 0.25 | 0.316 |
| *Prevotella* | 2.40 | 1.83 | 0.11 | 0.002 |
| Others | 45.00 | 56.06 | 2.86 | 0.044 |

Note：“Others” includes phyla, classes, orders, families or genera beyond the top 10. S0, the broilers were fed a basal diet; S3, the broilers were fed a basal diet supplementation with 0.6 % of BMFA. n=5.

**Table S3** Differential metabolites of the comparison group S3 vs S0

| **Rt^1^ (min)** | **Differential metabolites** | **m/z** | ***P*-value^2^** | **FC^3^** | **Score^4^** |
| --- | --- | --- | --- | --- | --- |
| 0.312^a^ | isoleucine | 130.08691 | 0.013 | 1.30 | 0.50 |
| 0.410 ^a^ | protocatechuic acid | 153.02238 | 0.029 | 0.27 | 0.50 |
| 0.624 ^a^ | pyrocatechol | 109.02887 | 0.040 | 0.29 | 1.00 |
| 1.009^b^ | hepoxilin a3 | 335.22307 | 0.008 | 0.27 | 0.72 |
| 1.796 ^a^ | dihydrojasmonic acid | 211.13402 | 0.006 | 0.38 | 0.99 |
| 2.152 ^a^ | indoleacetic acid | 174.05576 | 0.032 | 0.46 | 0.97 |
| 2.515 ^a^ | epicatechin | 289.07182 | 0.032 | 0.31 | 0.80 |
| 2.577 ^a^ | hydroxyoctanoic acid | 159.10242 | 0.045 | 3.88 | 0.53 |
| 2.987 ^b^ | sinapyl alcohol | 209.08165 | 0.007 | 0.29 | 0.68 |
| 3.252 ^a^ | kynurenic acid | 188.03542 | 0.036 | 0.52 | 0.98 |
| 3.784 ^a^ | xi-2,3-dihydro-2-oxo-1h-indole-3-acetic acid | 190.05080 | 0.042 | 0.13 | 0.88 |
| 3.842 ^a^ | indole-3-carbinol | 146.06087 | 0.031 | 0.28 | 0.81 |
| 4.326 ^b^ | N-acetyl-L-alanine | 130.05048 | 0.036 | 0.45 | 0.73 |
| 5.228 ^b^ | 1-octen-3-yl primeveroside | 467.21119 | 0.050 | 1.61 | 0.65 |
| 6.007 ^b^ | 4-hydroxyproline | 130.05064 | 0.025 | 0.59 | 0.64 |
| 9.037 ^a^ | L-lysine | 145.09775 | 0.007 | 0.52 | 0.60 |
| 33.387 ^c^ | tetrahydrocorticosterone | 370.00000 | 0.037 | 0.48 | 0.41 |

^1^ In the Rt (retention time) column, superscript a and b represent that samples were analyzed by LC-MS/MS in negative ion mode ([M-H] ^-^) or positive ion mode([M+H] ^+^), respectively. Additionally, superscript c represents that samples were detected via GC-MS.

^2^ A *P-*value of < 0 .05 was taken as statistically significant, calculated by Student *t*-test.

^3^ FC > 1.1 and FC < 0.9 indicated upregulated or downregulated metabolite, respectively.

^4^ The size of the node represents the matching degree of metabolite detected corresponding to the mass spectra of the standards in the in-house MS2 database (Biotree DB, Shanghai, China); the bigger the node size, the higher the degree.


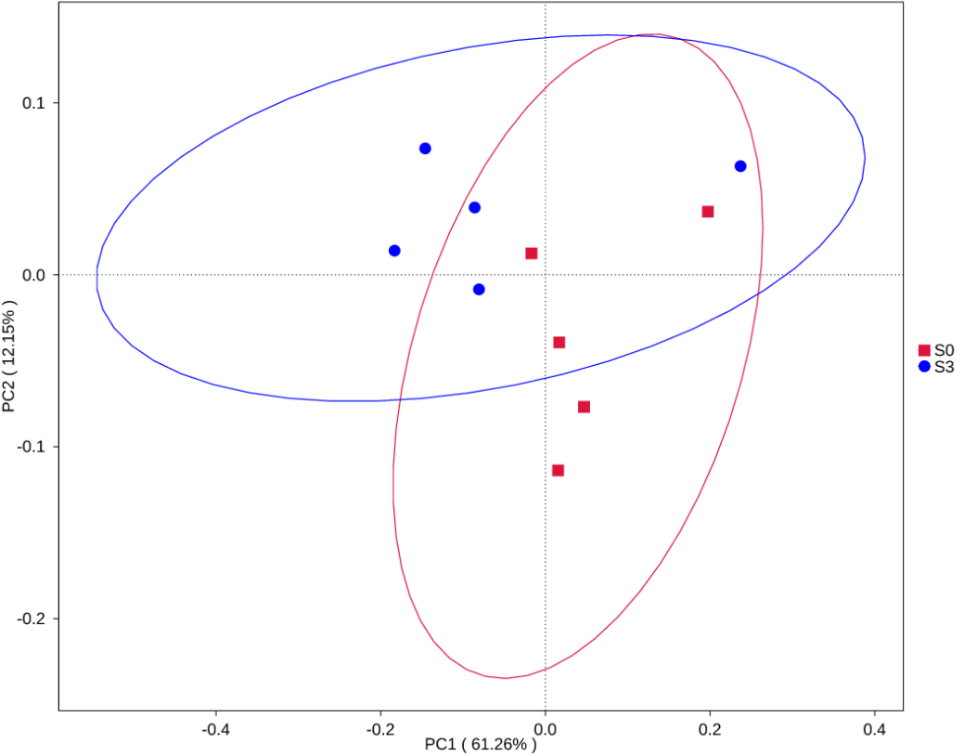


**Figure S1** Beta diversity analyses by PCoA

S0, the broilers were fed a basal diet; S3, the broilers were fed a basal diet supplementation with 0.6 % of BMFA. n=5.


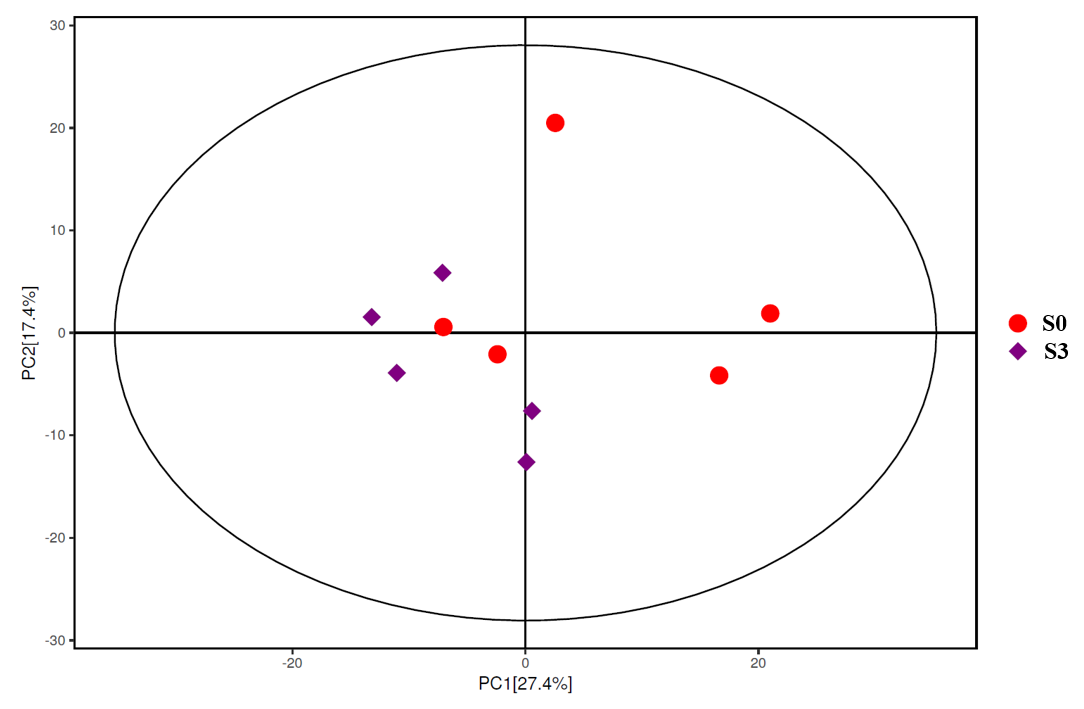


**(A)**


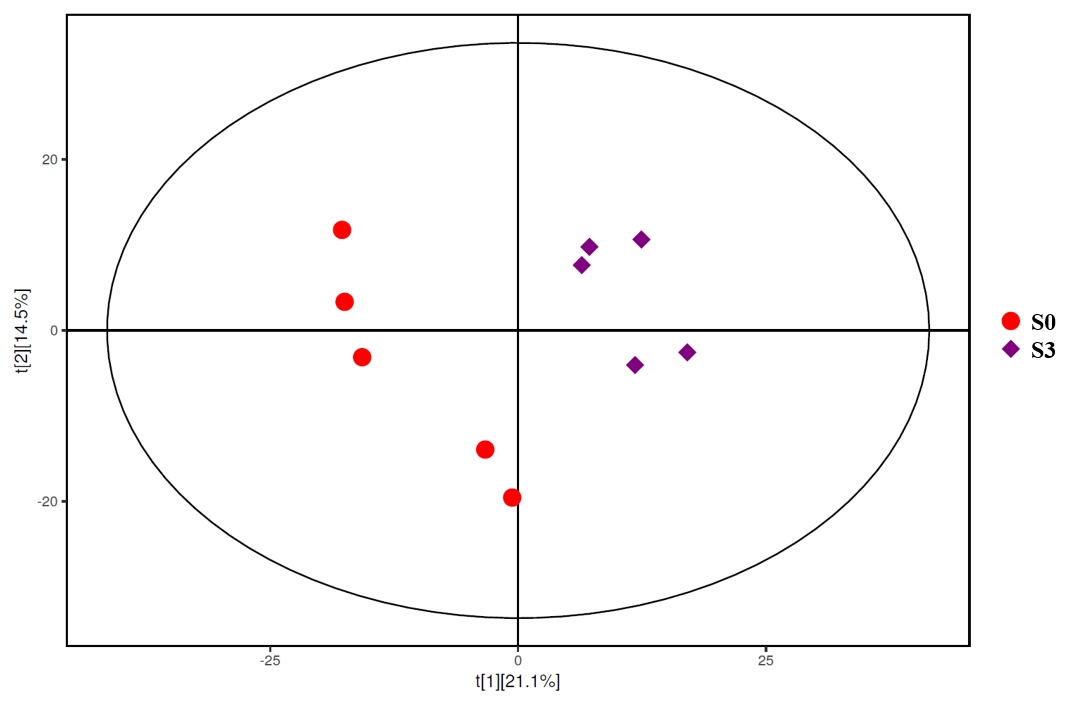
**(B)**

**Figure S2** Multivariate statistical analysis of the UPLC-MS/MS and GC-MS based metabolite data of the S0 and S3 groups: (A) PCA scores plot; and (B) PLS-DA scores plot. S0, the broilers were fed a basal diet; S3, the broilers were fed a basal diet supplementation with 0.6 % of BMFA. n=5.


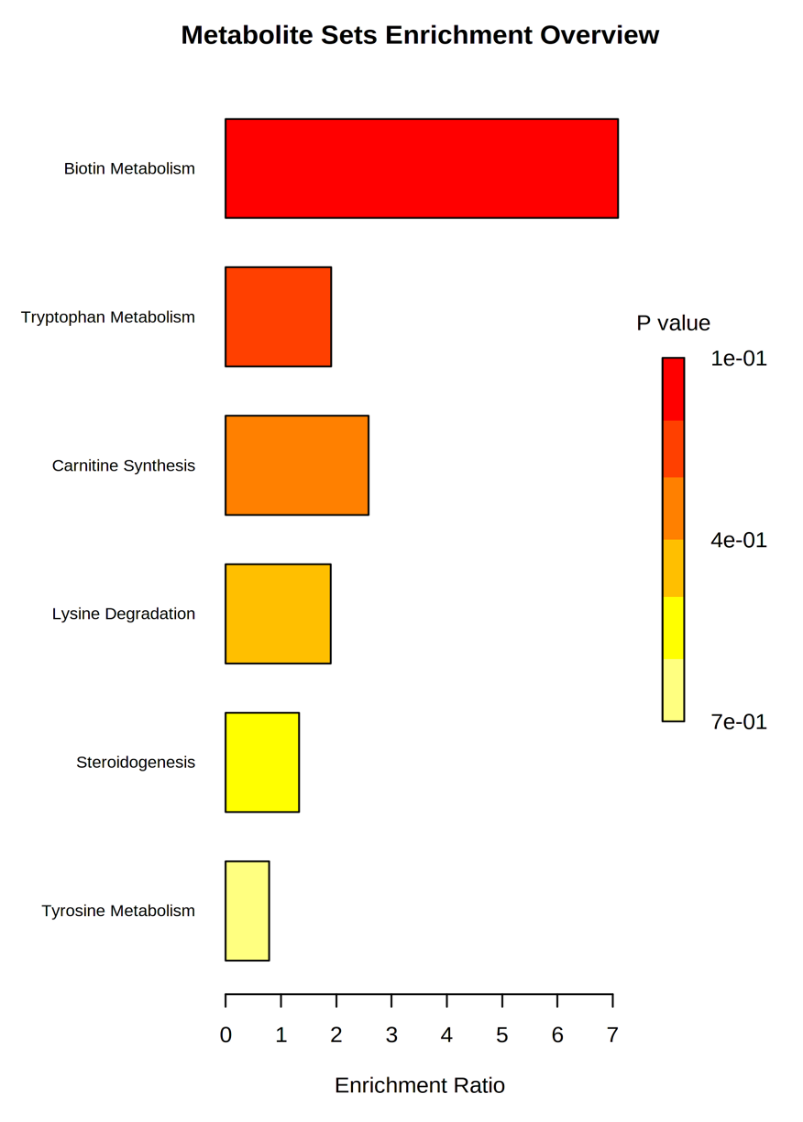


**Figure S3** Pathway enrichment analysis of differential metabolites between the S0 and S3. S0, the broilers were fed a basal diet; S3, the broilers were fed a basal diet supplementation with 0.6 % of BMFA. n=5.
